# Supplementary material for: Structure of a cyanobacterial photosystem I tetramer revealed by cryo-electron microscopy
Source: Nat Commun. 2019 Oct 30;10:4929. doi: 10.1038/s41467-019-12942-8 (PMC6821847; doi:10.1038/s41467-019-12942-8)
Supplement: Supplementary file 1 — Supplementary Information [file 41467_2019_12942_MOESM1_ESM.pdf]

Supplementary Information

Structure of a cyanobacterial photosystem I tetramer revealed by cryo-electron microscopy

Koji Kato, Ryo Nagao, Tian-Yi Jiang et al.

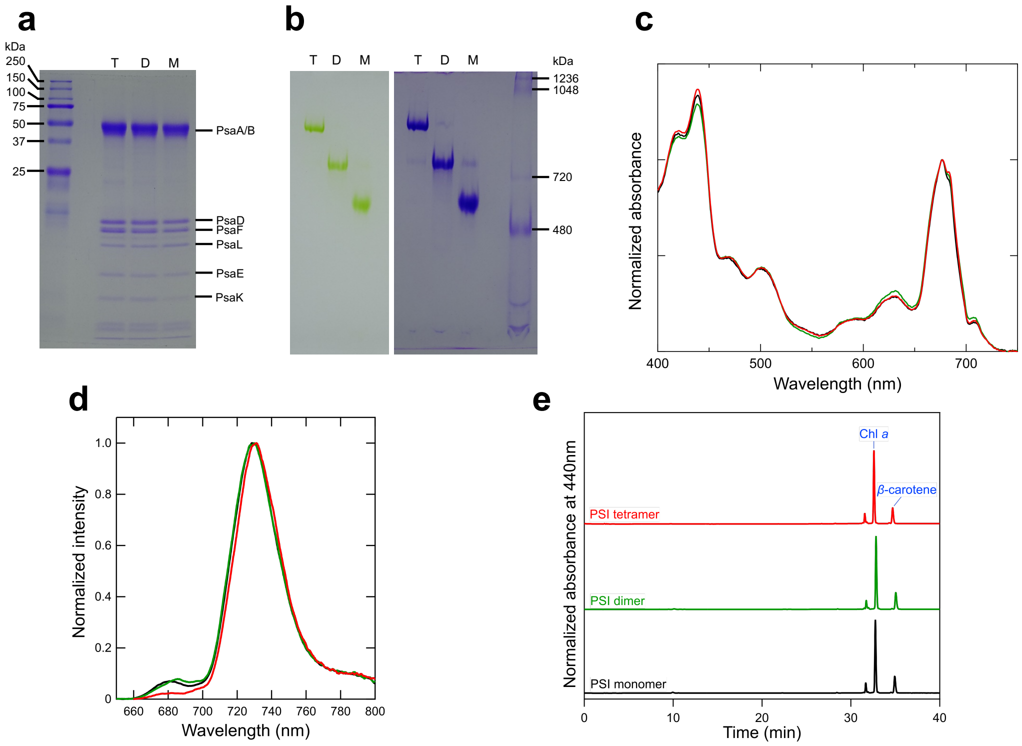

Supplementary Fig. 1. Biochemical and spectroscopic characterizations of the PSI core complexes. a, SDS-PAGE analysis of the PSI cores. Letters T, D and M in each lane indicate the PSI tetramer, dimer and monomer, respectively. **b,** Clear native-PAGE analysis of the PSI cores. The gels were shown either directly without staining (left side) or stained with CBB (right side). Letters T, D and M in each lane indicate the PSI tetramer, dimer and monomer, respectively. **c,** Absorption spectra measured at 77 K. The spectra of the PSI tetramer, dimer and monomer are depicted in red, green and black, respectively, and were normalised by the Qy peak of Chl *a* at 677 nm. **d,** Normalised fluorescence spectra excited at 445 nm. The spectra of the PSI tetramer, dimer and monomer are depicted in red, green and black, respectively. **e,** HPLC analyses of the PSI core pigments monitored at 440 nm. The chromatograms of the PSI tetramer, dimer and monomer are depicted in red, green and black, respectively, and were normalised by the peak of Chl *a*.

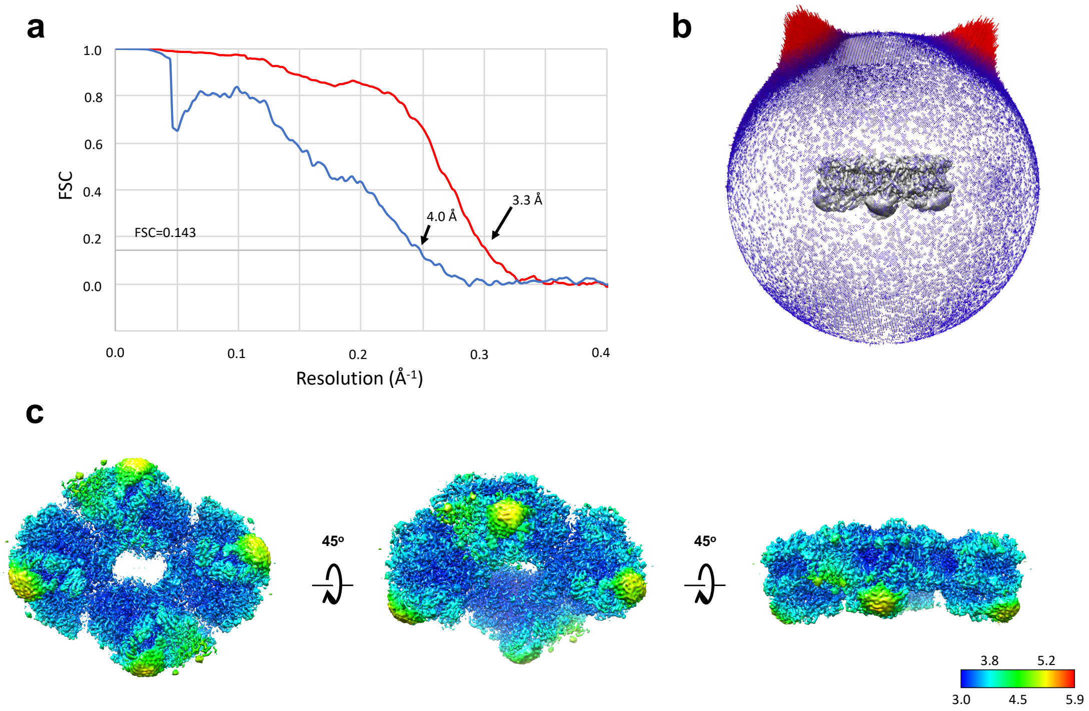

Supplementary Fig. 2. Evaluation of the cryo-EM map quality. **a,** Fourier shell correlation curves of the PSI tetramer (red) and dimer (blue) calculated between independently refined half maps used for the structure reconstructions. **b,** Angular distribution of the particles used for reconstruction of PSI tetramer complex. Each cylinder represents one view and the height of the cylinder is proportional to the number of particles for that view. **c,** Local resolution maps of the PSI tetramer.

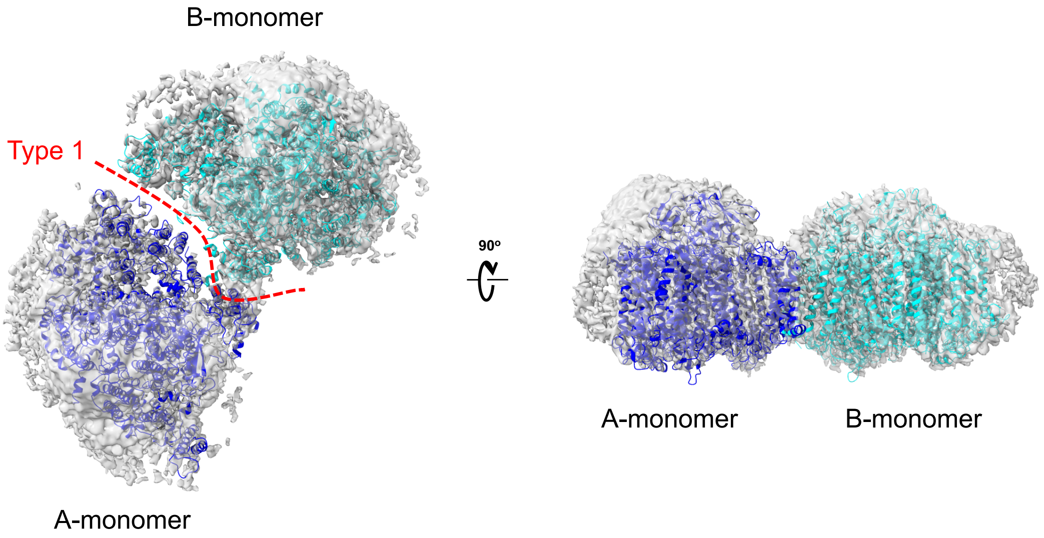

Supplementary Fig. 3. Overall structure of a PSI dimer. The 3D cryo-EM density maps of the PSI dimer superimposed with a cartoon model of the PSI dimer, with a view along the membrane normal from the stromal side (Left side) and its side view (Right side).

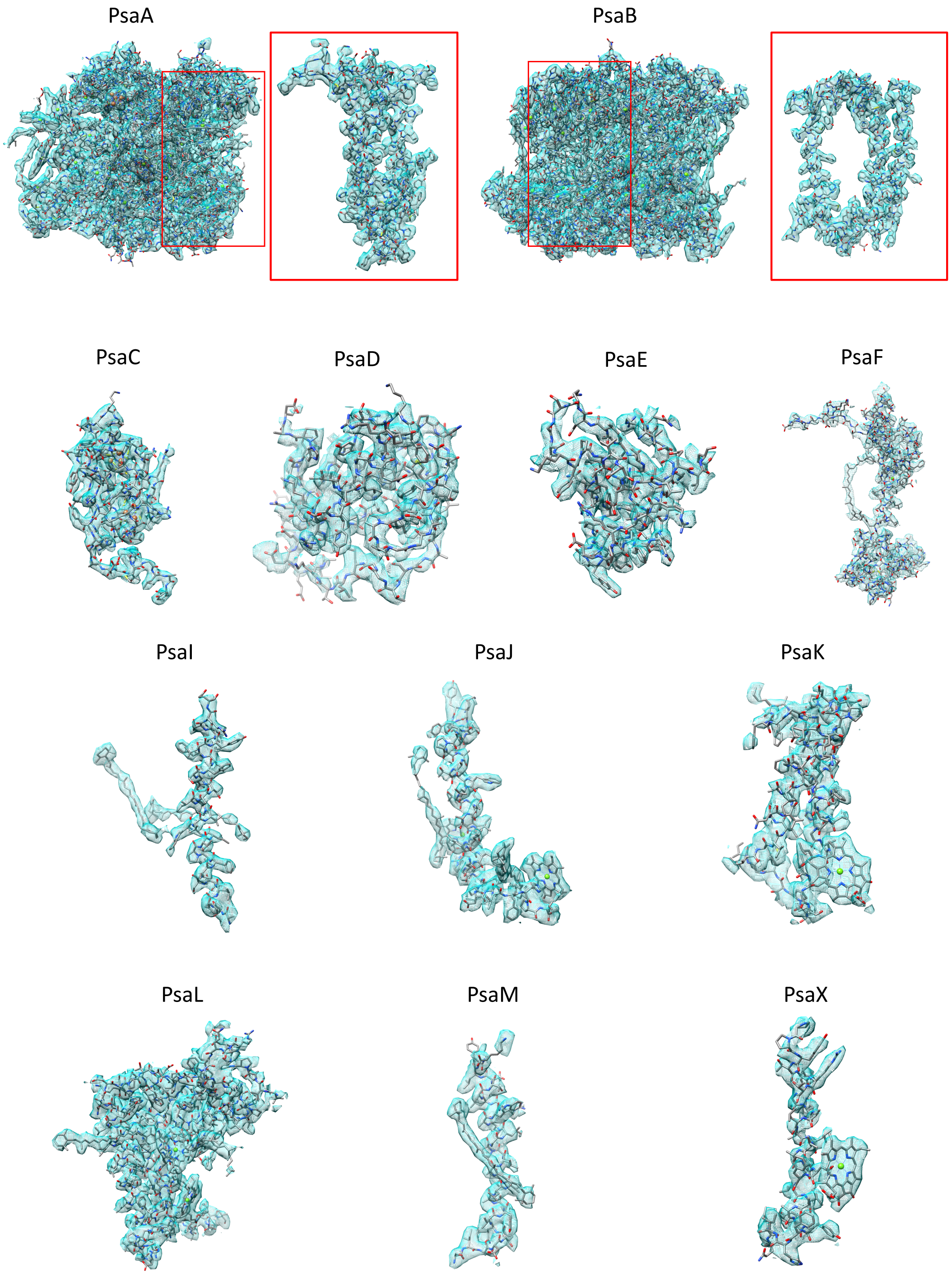

Supplementary Fig. 4. Cryo-EM density maps and structures of the PSI core subunits. The densities for each subunit of PSI are shown as blue meshes and the corresponding models are shown as gray sticks. Red boxes indicate the enlarged views for a part of the individual PsaA and PsaB subunits.

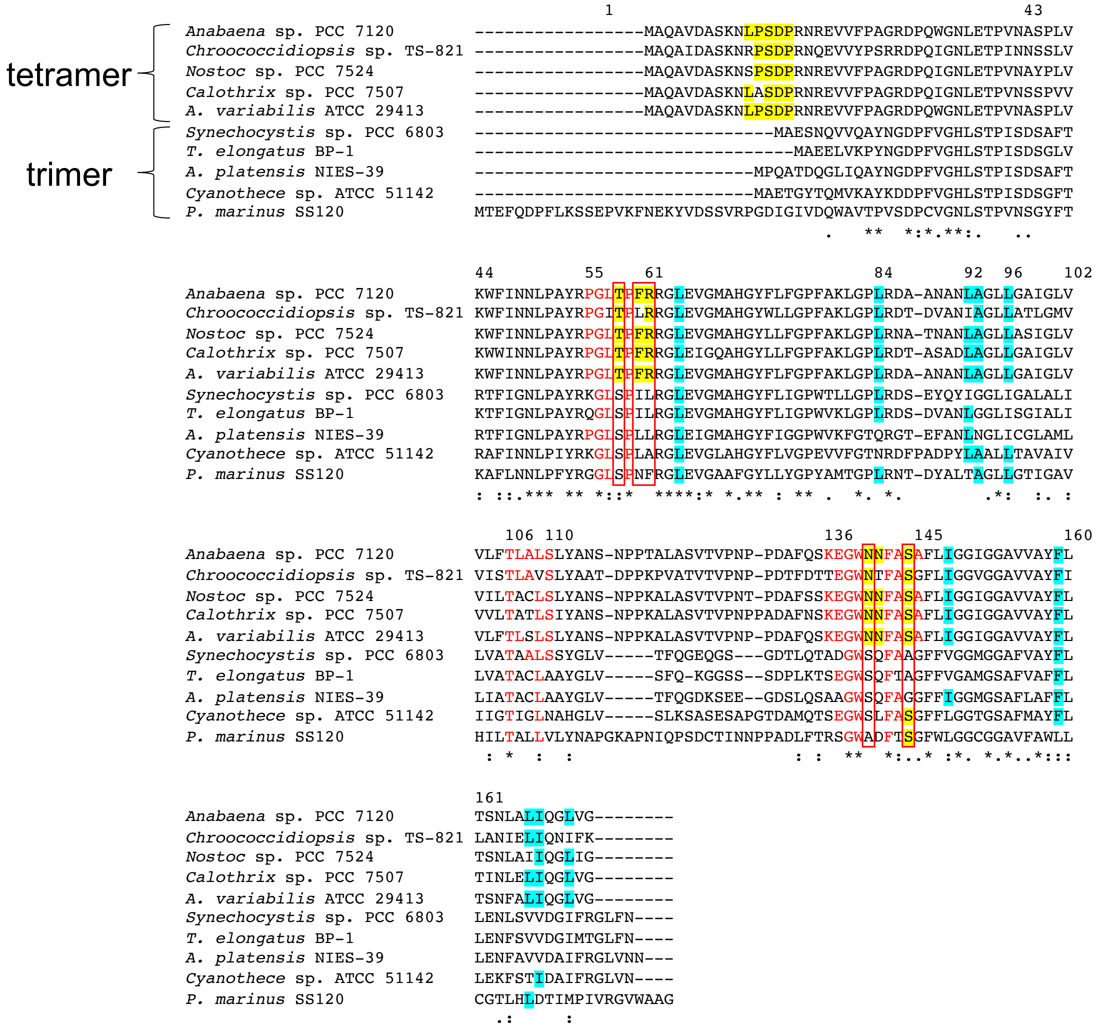

Supplementary Fig. 5. Multiple sequence alignment (CLUSTALW) of the cyanobacterial PsaL subunit. The amino acid residues for the contact regions in Type 1 interface, the steric hindrance regions for the trimer and the regions possessing both of these properties are highlighted in blue, red and yellow, respectively. The red boxes show the bulky side chains in the steric hinderance region. The cyanobacterial species shown are *Anabaena* sp. PCC 7120, *Chroococcidiopsi* sp. TS-821, *Nostoc* sp. PCC 7524, *Calothrix* sp. PCC 7507, *Anabaena variabilis* ATCC 29413, *Synechocystis* sp. PCC 6803, *Thermosynechococcus elongatus* BP-1, *Arthrospira platensis* NIES-39, *Cyanothece* sp. ATCC 51142 and *Prochlorococcus marinus* SS120.

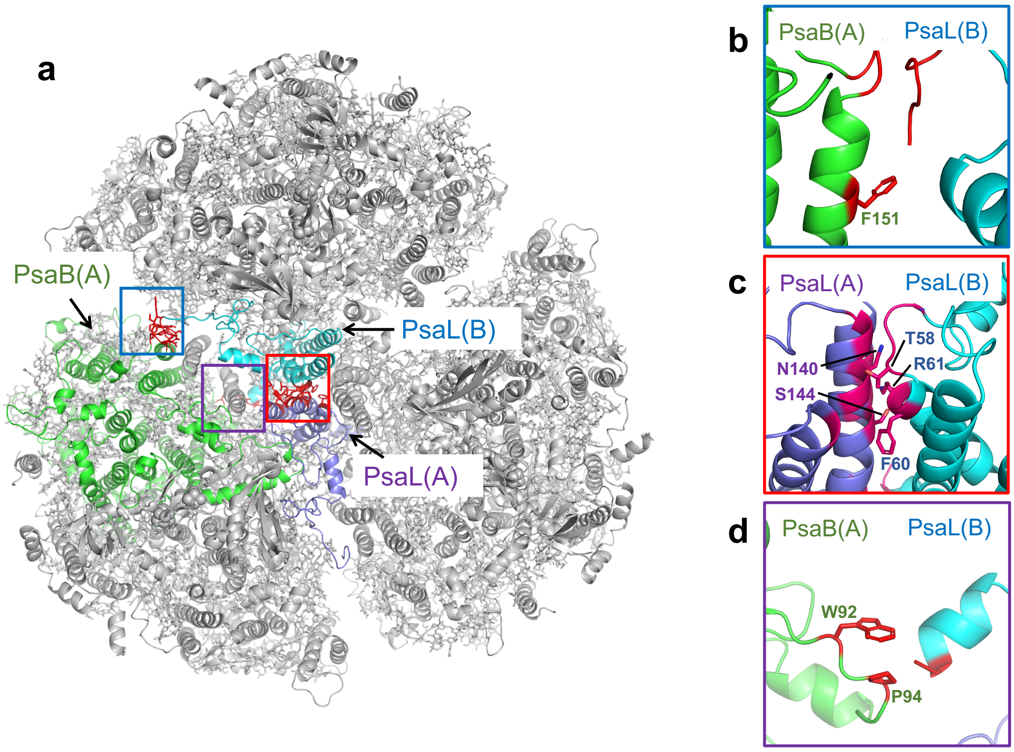

Supplementary Fig. 6. Modelling of the *Anabaena* PSI trimer. a, Superposition of an *Anabaena* PSI monomer with the trimeric structure of PSI from *T. elongatus*. The regions having steric hindrances are coloured in red. PsaB in A-monomer, PsaL in A-monomer and in B-monomer are depicted in green, purple and cyan, respectively. **b-d,** Close-up views of the steric hindrance in the N terminal (**b**), middle (**c**) and C terminal (**d**) regions of PsaL.

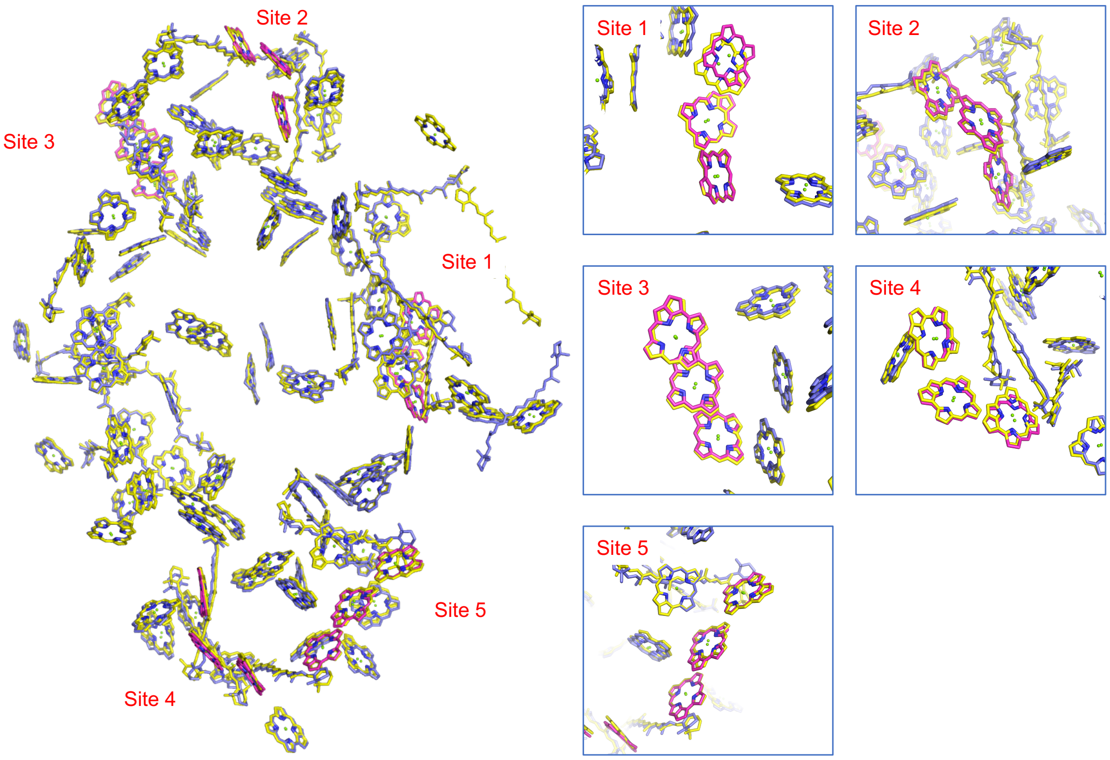

Supplementary Fig. 7. Superposition of the pigment molecules of the PSI tetramer with those of the PSI trimer. The pigment molecules of the PSI tetramer and trimer are depicted in purple and yellow, respectively. Red colored Chls indicate the triple Chls in the tetramer from Site 1 to 5. The close-up views of Site 1 to 5 Chls are shown in the right panels.

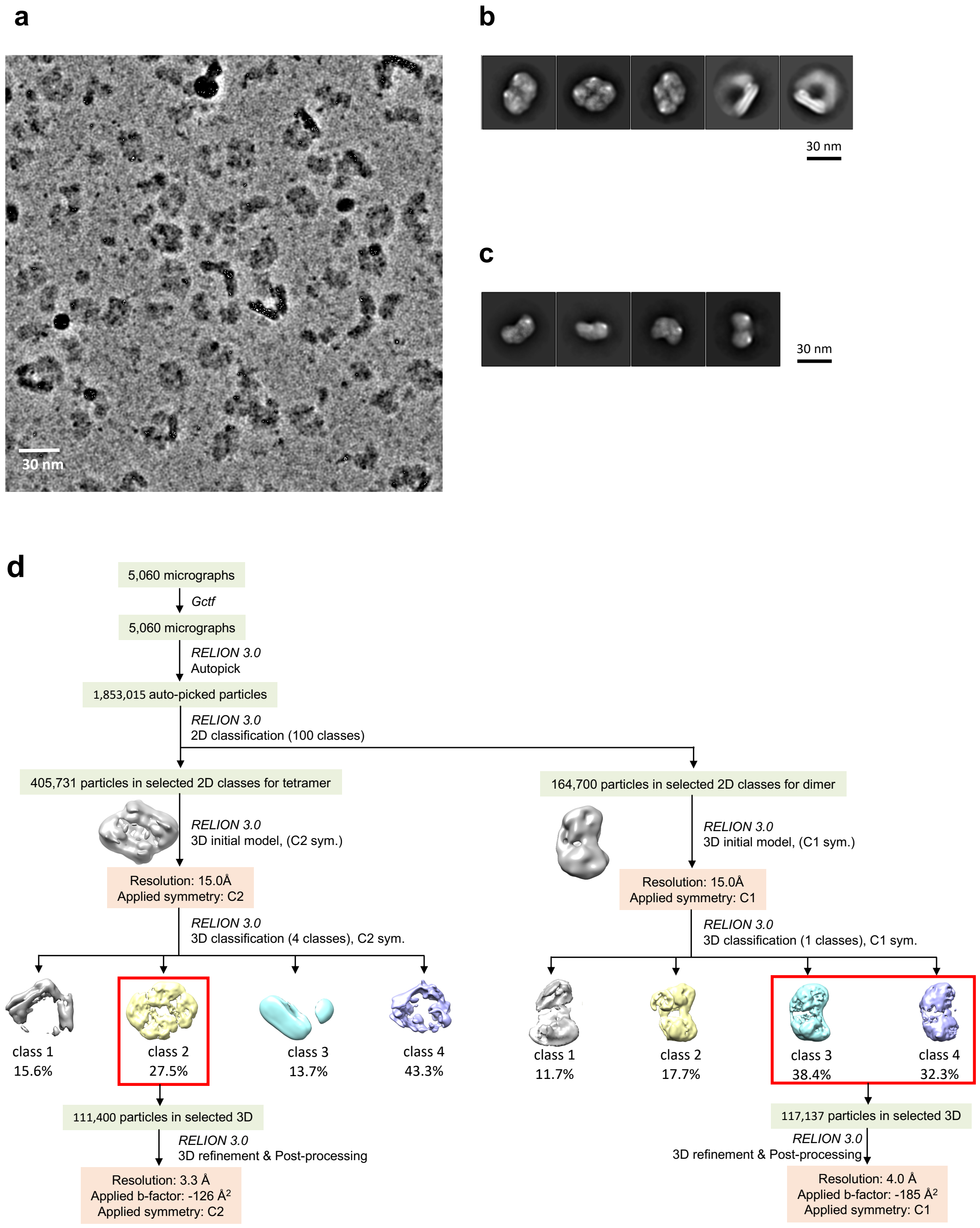

Supplementary Fig. 8. Cryo-EM data collection and processing. a, A representative cryo-EM micrograph of the *Anabaena* PSI tetramer. **b,** Representative 2D classes of the PSI tetramer particles. **c,** Representative 2D classes of the PSI dimer particles. **d,** A schematic flowchart showing the classification scheme for the PSI tetramer and dimer complexes. The PSI tetramer structure and dimer structure were reconstructed at 3.3 Å resolution from 111,400 particles and 4.0 Å resolution from 117,137 particles, respectively. See Methods section for more details.

Supplementary Table 1. Statistics of data collection, processing and refinement.

Complex	PSI tetramer	PSI dimer
PDB ID	6JEO	-
EMDB ID	EMD-9807	EMD-9877
Data collection and processing		
Microscope	FEI Titan Krios G2	
Detector	Falcon 3EC direct electron detector	
Magnification	59000	
Voltage (kV)	300	
Defocus range (μm)	-0.6 to -0.8	
Pixel size (Å)	1.12	
Total electron dose ($e^-/Å^2$)	40	
Exposure time (s)	2.0	
Number of frames per image	26	
Number of micrographs	5159	
Initial particle images (no.)	1853015	
Final particle images (no.)	111400	117137
Map resolution (Å)	3.3	4.0
Applied b-factor ($Å^2$)	-126	-185
Applied symmetry	C2	C1
Refinement		
Initial Model used (PDB code)	1JB0	-
Model resolution (Å)	3.3	-
FSC threshold	0.143	-
No. of atoms		
Protein	68908	-
Ligand	25668	-
B factors ($Å^2$)		
Protein	31.6	-
Ligand	24.0	-
R.m.s deviations		
Bond lengths (Å)	0.029	-
Bond angles (°)	2.295	-
Validation		
MolProbity score	1.75	-
Clashscore	7.49	-
Poor rotamers (%)	0.24	-
EMRinger score	4.38	-
Ramachandran plot		
Favored (%)	95.18	-
Allowed (%)	4.77	-
Disallowed (%)	0.05	-

Supplementary Table 2. Cofactors in each monomer unit of the PSI tetramer.

Protein	Chorophyll	Carotenoid	Lipid	Others
PsaA	45 Chl *a*<br>1 Chl *a′*	6 BCR	2 LHG	1 [4Fe-4S] cluster,<br>1 phylloquinone
PsaB	41 Chl *a*	7 BCR	1 LMG<br>1 LHG	1 phylloquinone
PsaC				2 [4Fe-4S] cluster
PsaD				
PsaE				
PsaF	1 Chl *a*	1 BCR	1 DGD	
PsaI		2 BCR		
PsaJ	2 Chl *a*	3 BCR		
PsaK	1 Chl *a*			
PsaL	3 Chl *a*	2 BCR		
PsaM		1 BCR		
PsbX	1 Chl *a*			
Total	95	22	5	5

BCR, β-carotene; LMG, distearoylmonogalactosyl diglyceride; LHG, dipalmitoylphosphatidyl glycerol; DGD, digalactosyldiacyl glycerol.

Supplementary Table 3. Correspondence of numbering of pigments described in the text with those in the PDB file for each monomers in PSI tetramer.

	PSI monomers	A-monomer	B-monomer	A'-monomer	B'-monomer
**Subunits**	**Chls in the text**	**PDB No. (Chain ID)**	**PDB No. (Chain ID)**	**PDB No. (Chain ID)**	**PDB No. (Chain ID)**
**PsaA**	813	812 (aA)	812 (bA)	812 (cA)	812 (dA)
	814	813 (aA)	813 (bA)	813 (cA)	813 (dA)
	821	820 (aA)	820 (bA)	820 (cA)	820 (dA)
	823	822 (aA)	822 (bA)	822 (cA)	822 (dA)
	824	823 (aA)	823 (bA)	823 (cA)	823 (dA)
	834	833 (aA)	833 (bA)	833 (cA)	833 (dA)
	835	834 (aA)	834 (bA)	834 (cA)	834 (dA)
	846	843 (aA)	202 (bL)*	843 (cA)	202 (dL) *
**PsaB**	810	201 (aL)*	811 (bB)	201 (cL)*	811 (dB)
	812	812 (aB)	813 (bB)	812 (cB)	813 (dB)
	813	813 (aB)	814 (bB)	813 (cB)	814 (dB)
	820	820 (aB)	821 (bB)	820 (cB)	821 (dB)
	834	834 (aB)	835 (bB)	834 (cB)	835 (dB)
	835	835 (aB)	836 (bB)	835 (cB)	836 (dB)
	836	836 (aB)	837 (bB)	836 (cB)	837 (dB)
	**Carotenoids in the text**				
**PsaA**	849	846 (aA)	102 (bK)*	846 (cA)	102 (dK)*
	850	847 (aA)	845 (bA)	847 (cA)	845 (dA)
	851	848 (aA)	846 (bA)	848 (cA)	846(dA)
	852	849 (aA)	847 (bA)	849 (cA)	847 (dA)
**PsaB**	843	843 (aB)	844 (bB)	843 (cB)	844 (dB)
	844	844 (aB)	845 (bB)	844 (cB)	845 (dB)
	847	847 (aB)	848 (bB)	847 (cB)	848 (dB)
**PsaI**	101	101 (aI)	101 (bI)	101 (cI)	101 (dI)
	102	201 (aL)*	203 (bL)*	201 (cL)*	203 (dL)*

*Chains in the adjacent unit.
